# Correction: Colomba et al. Haemodynamic Forces: Emerging Markers of Ventricular Remodelling in Multiple Myeloma Cardiovascular Baseline Risk Assessment. *Cancers* 2024, *16*, 3081

**DOI:** 10.3390/cancers16233891

**Published:** 2024-11-21

**Authors:** Anna Colomba, Anna Astarita, Giulia Mingrone, Lorenzo Airale, Cinzia Catarinella, Fabrizio Vallelonga, Dario Leone, Marco Cesareo, Arianna Paladino, Sara Bringhen, Francesca Gay, Gianni Pedrizzetti, Franco Veglio, Alberto Milan

**Affiliations:** 1 Candiolo Cancer Institute, FPO—IRCCS, 10060 Candiolo, TO, Italy; anna.colomba@unito.it (A.C.); giulia.mingrone@unito.it (G.M.); fabrizio.vallelonga@ircc.it (F.V.); ariannapaladino@hotmail.it (A.P.); alberto.milan@unito.it (A.M.); 2 Department of Medical Sciences, University of Turin, 10126 Turin, Italy; 3 Hypertension Unit, Department of Medical Sciences, Division of Internal Medicine, AOU Città della Salute e della Scienza University Hospital, 10126 Turin, Italy; anna.astarita@unito.it (A.A.); lorenzo.airale@unito.it (L.A.); cinzia.catarinella@unito.it (C.C.); marcor.cesareo@gmail.com (M.C.); franco.veglio@unito.it (F.V.); 4 SSD Clinical Trial in Oncoematologia e Mieloma Multiplo, Division of Hematology, AOU Città della Salute e della Scienza University Hospital, 10126 Turin, Italy; sarabringhen@yahoo.com (S.B.); francesca.gay@unito.it (F.G.); 5 Department of Molecular Biotechnology and Health Sciences, Division of Hematology, University of Torino, 10124 Turin, Italy; 6 Department of Engineering and Architecture, University of Trieste, 34127 Trieste, Italy; gianni.pedrizzetti@dia.units.it

## Error in Affiliation

There was an error in the original publication [1]. The affiliation for Dario Leone and for the co-authors Anna Colomba, Giulia Mingrone, Fabrizio Vallelonga, Arianna Paladino and Alberto Milan was incorrectly written.

It is not the following:

1. Department of Medical Sciences, Division of Internal, Medicine, Candiolo Cancer Institute FPO-IRCCS, University of Turin, Str.Prov.le 142, km 3,95, 10060 Candiolo, Italy.

The correct version is as follows: 

1. Candiolo Cancer Institute, FPO—IRCCS, 10060 Candiolo, TO, Italy; 

2. Department of Medical Sciences, University of Turin, 10126 Turin, Italy

## Missing Funding

In the original publication [1], the funder “Italian Ministry of Health”, “Ricerca Corrente 2024” to “Dario Leone” was not included. Also, the correct acknowledgment for LILT—Metropolitan City of Turin was not specified. The correct Funding is:

Dario Leone acknowledges partial support by the Italian Ministry of Health with grant: Ricerca Corrente 2024. Arianna Paladino has received a grant for research from LILT—Metropolitan City of Turin (Lega Italiana Lotta per i Tumori). Gianni Pedrizzetti acknowledges partial support by the Italian Ministry of Education and Research with grant: PRIN 2022AJT27Y, provided through the University of Trieste (CUP J53D23002030006). 

The authors apologize for any inconvenience caused and state that the scientific conclusions are unaffected. This correction was approved by the Academic Editor. The original publication has also been updated.

All authors have read and agreed to the published version of the manuscript.
