# Supplementary material for: Haemodynamic Forces: Emerging Markers of Ventricular Remodelling in Multiple Myeloma Cardiovascular Baseline Risk Assessment
Source: Cancers (Basel). 2024 Sep 4;16(17):3081. doi: 10.3390/cancers16173081 (PMC11393942; doi:10.3390/cancers16173081)
Supplement: Supplementary file 1 [file cancers-16-03081-s001.zip › cancers-3162512-supplementary.pdf]

Supplementary Materials

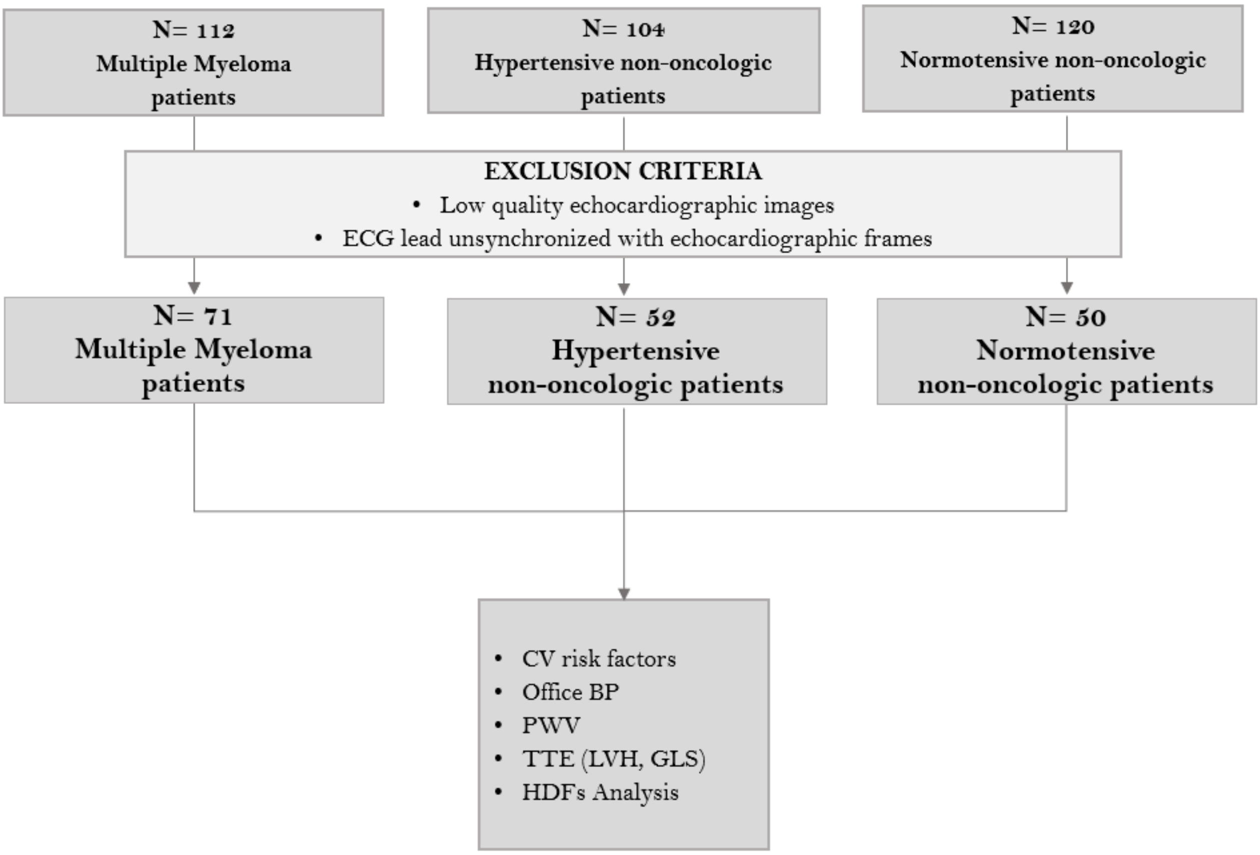

Figure S1: *Study protocol. ECG: Electrocardiogram, CV: cardiovascular, BP: blood pressure, PWV: Pulse Wave Velocity, TTE: Trans Thoracic Echocardiography, LVH: Left Ventricular Hypertrophy, GLS: Global Longitudinal Strain, HDFs: Hemodynamic Forces.*

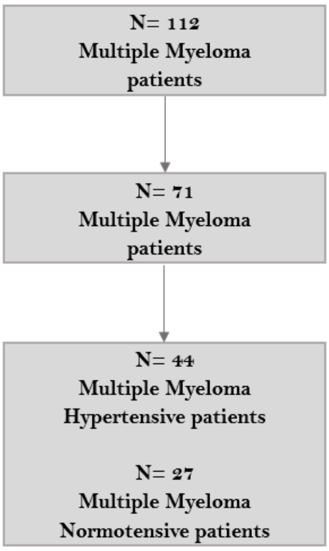

Figure S2: *study protocol: separation of MM patients in 2 cohorts.*

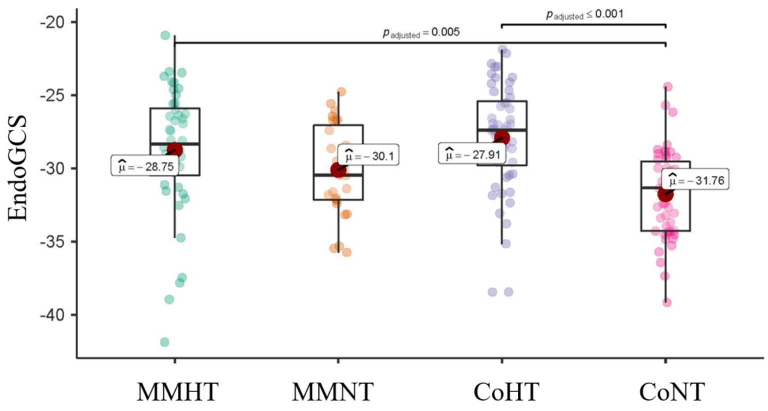
EndoGCS
-20
-25
-30
-35
-40
$p_{adjusted} = 0.005$
$p_{adjusted} \leq 0.001$
$\hat{\mu} = -28.75$
$\hat{\mu} = -30.1$
$\hat{\mu} = -27.91$
$\hat{\mu} = -31.76$
MMHT
MMNT
CoHT
CoNT

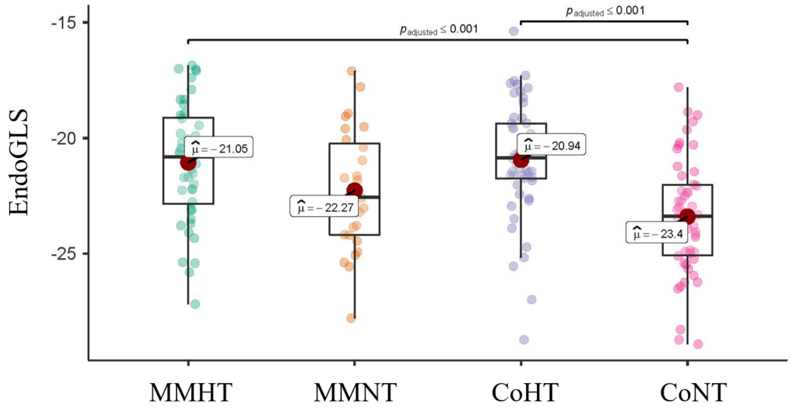
EndoGLS
-15
-20
-25
$p_{adjusted} \leq 0.001$
$p_{adjusted} \leq 0.001$
$\hat{\mu} = -21.05$
$\hat{\mu} = -22.27$
$\hat{\mu} = -20.94$
$\hat{\mu} = -23.4$
MMHT
MMNT
CoHT
CoNT

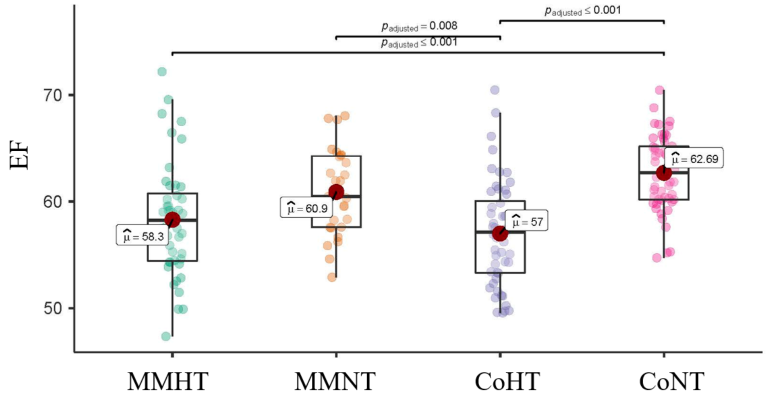
EF
70
60
50
$p_{adjusted} \leq 0.001$
$p_{adjusted} = 0.008$
$p_{adjusted} \leq 0.001$
$\hat{\mu} = 58.3$
$\hat{\mu} = 60.9$
$\hat{\mu} = 57$
$\hat{\mu} = 62.69$
MMHT
MMNT
CoHT
CoNT

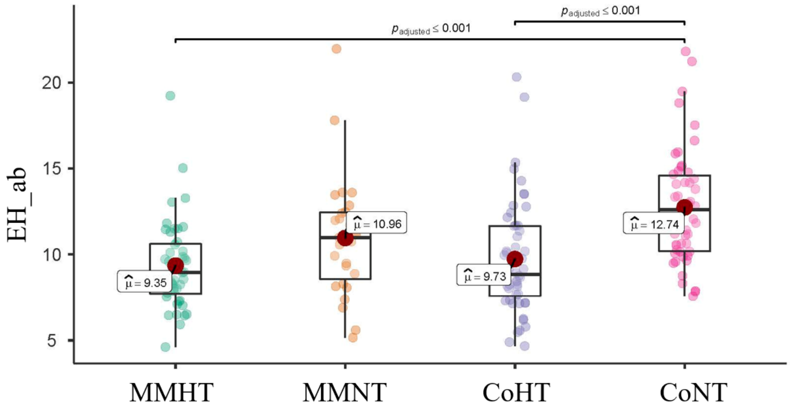
EH_ab
20
15
10
5
$p_{adjusted} \leq 0.001$
$p_{adjusted} \leq 0.001$
$\hat{\mu} = 9.35$
$\hat{\mu} = 10.96$
$\hat{\mu} = 9.73$
$\hat{\mu} = 12.74$
MMHT
MMNT
CoHT
CoNT

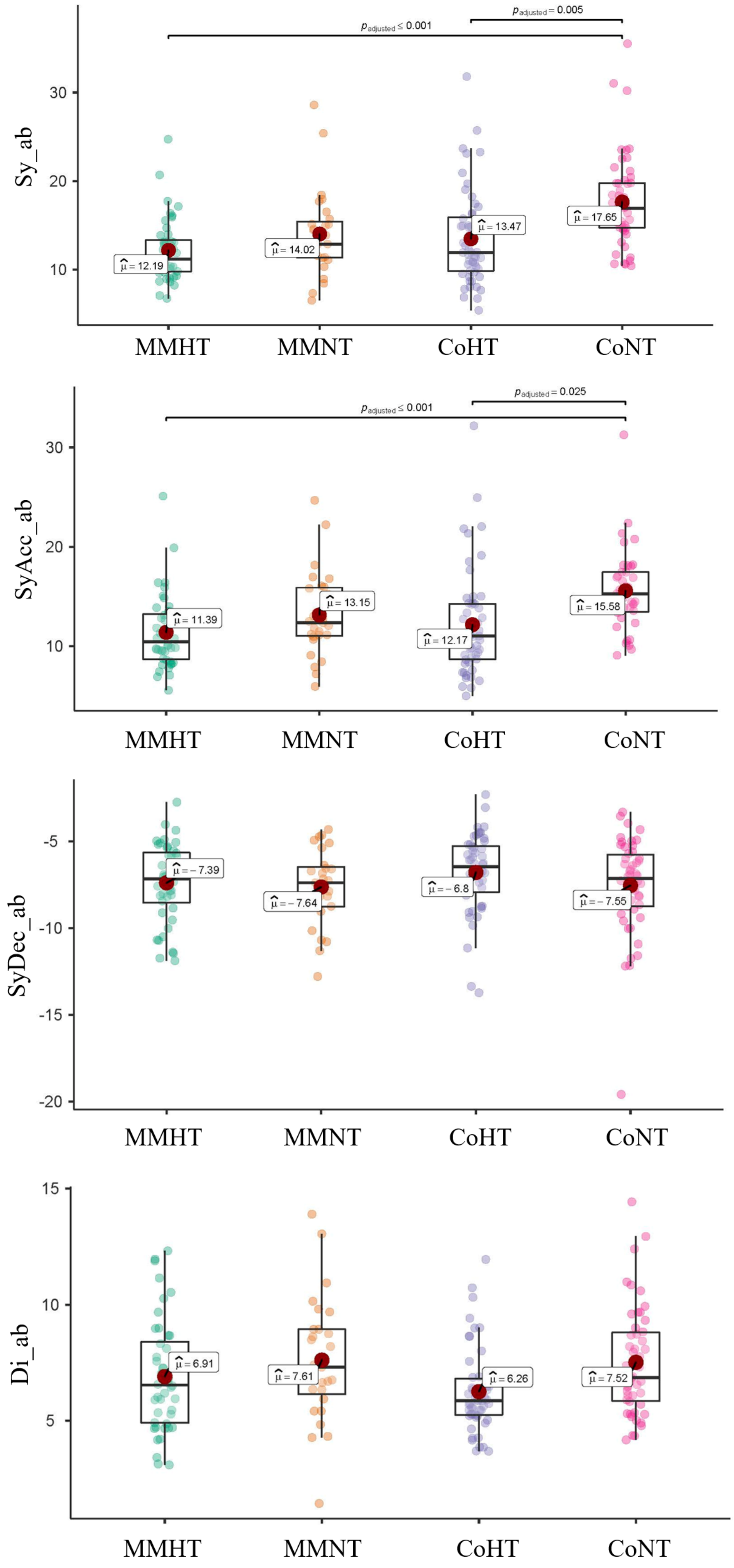

p_adjusted ≤ 0.001
p_adjusted = 0.005
Sy_ab
30
20
10
μ̂ = 12.19
μ̂ = 14.02
μ̂ = 13.47
μ̂ = 17.65
MMHT
MMNT
CoHT
CoNT
p_adjusted ≤ 0.001
p_adjusted = 0.025
SyAcc_ab
30
20
10
μ̂ = 11.39
μ̂ = 13.15
μ̂ = 12.17
μ̂ = 15.58
MMHT
MMNT
CoHT
CoNT
SyDec_ab
-5
-10
-15
-20
μ̂ = − 7.39
μ̂ = − 7.64
μ̂ = − 6.8
μ̂ = − 7.55
MMHT
MMNT
CoHT
CoNT
Di_ab
15
10
5
μ̂ = 6.91
μ̂ = 7.61
μ̂ = 6.26
μ̂ = 7.52
MMHT
MMNT
CoHT
CoNT

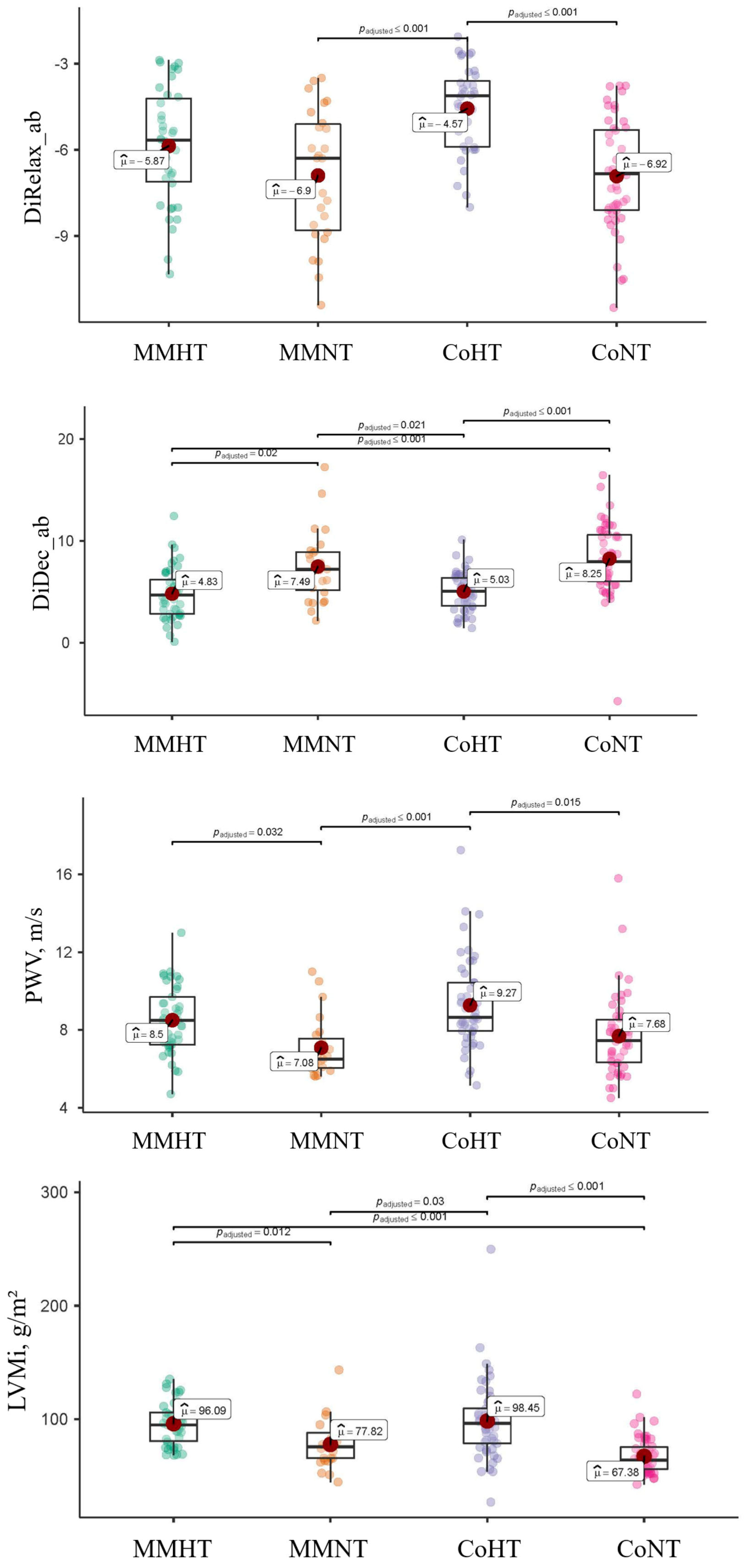

Figure S3: *box plots from comparative analysis.*

MMHT: Multiple myeloma Hypertensive Group, MMNT: Multiple Myeloma

Normotensive Group, CoHT: Non-oncologic Hypertensive Group, CoNT: Non-oncologic Normotensive Group, EF: Ejection Fraction, GCS: Global Circumferential Strain, GLS: Global Longitudinal Strain, ab: apico-basal, EH: Entire Heartbeat, Sy: systole, SyAcc: Systolic Acceleration, SyDec: Systolic Deceleration, Di: Diastole, DiRelax: Diastolic Relaxation, DiDec: Diastolic Deceleration, PWV: Pulse Wave Velocity, LVMi: Left Ventricular Mass index.
